# *Premna* Species in Vietnam: Essential Oil Compositions and Mosquito Larvicidal Activities

**DOI:** 10.3390/plants9091130

**Published:** 2020-08-31

**Authors:** Nguyen Huy Hung, Le Thi Huong, Nguyen Thanh Chung, Nguyen Cong Truong, Do Ngoc Dai, Prabodh Satyal, Thieu Anh Tai, Vu Thi Hien, William N Setzer

**Affiliations:** 1 Center for Advanced Chemistry, Institute of Research and Development, Duy Tan University, 03 Quang Trung, Da Nang 550000, Vietnam; 2 Department of Pharmacy, Duy Tan University, 03 Quang Trung, Da Nang 550000, Vietnam; anhtai0808qn@gmail.com; 3 School of Natural Science Education, Vinh University, 182 Le Duan, Vinh City 43000, Nghe An Province, Vietnam; lehuong223@gmail.com; 4 Graduate University of Science and Technology, Vietnam Academy of Science and Technology, 18-Hoang Quoc Viet, Cau Giay, Hanoi 10000, Vietnam; chungpuhoat@gmail.com (N.T.C.); daidn23@gmail.com (D.N.D.); 5 Faculty of Agriculture, Forestry and Fishery, Nghe An College of Economics, 51-Ly Tu Trong, Vinh City 43000, Nghe An Province, Vietnam; congtruong777@gmail.com; 6 Aromatic Plant Research Center, 230 N 1200 E, Suite 102, Lehi, UT 84043, USA; psatyal@aromaticplant.org; 7 Faculty of Hydrometerology, Ho Chi Minh City University of Natural Resources and Environment, Ho Chi Minh City 70000, Vietnam; hiensphoa@gmail.com; 8 Department of Chemistry, University of Alabama in Huntsville, Huntsville, AL 35899, USA

**Keywords:** Lamiaceae, *Aedes aegypti*, sesquiterpene hydrocarbons

## Abstract

Essential oils have emerged as viable alternatives to synthetic insecticides for control of mosquito-borne pathogens. The leaf essential oils of eight species of *Premna* (Lamiaceae) growing in central Vietnam have been obtained by hydrodistillation and analyzed by gas chromatography–mass spectrometry. Sesquiterpene hydrocarbons dominated most of the *Premna* essential oils, with the notable exception of *Premna* *mekongensis* from Ngoc Linh Nature Reserve, which had α-pinene as the major component. Larvicidal activities against *Aedes aegypti* have been determined and all of the *Premna* essential oils showed larvicidal activity with 24-h LC_50_ < 65 μg/mL. The leaf essential oils of *Premna* *cambodiana* from Chu Mom Ray National Park and *Premna* *mekongensis* from Ngoc Linh Nature Reserve showed the best larvicidal activities with 24-h LC_50_ of 16.8 and 18.0 μg/mL, respectively. The essential oil compositions and larvicidal activities of *P. cambodiana*, *Premna flavescens*, *Premna* *maclurei*, *P. mekongensis*, and *Premna* *puberula* are reported for the first time. Although the larvicidal activities of *Premna* leaf essential oils are promising, the essential oil yields are relatively low (0.10–0.25%).

## 1. Introduction

Mosquito-borne infectious diseases have been a persistent problem in Vietnam. Dengue fever and dengue hemorrhagic fever are especially problematic and chikungunya fever is an emerging threat in the country [1,2]. *Aedes aegypti* (L.) (Diptera: Culicidae), the yellow fever mosquito, and *Aedes albopictus* (Skuse) (Diptera: Culicidae), the Asian tiger mosquito, are important vectors of several viral pathogens, including dengue fever virus [3], yellow fever virus [4], chikungunya fever virus [5], and possibly Zika virus [6]. *Culex quinquefasciatus* Say (Diptera: Culicidae), the southern house mosquito, is a vector of lymphatic filariasis [7] as well as several arboviruses such as West Nile virus and St. Louis encephalitis virus [8], and possibly Zika virus [9].

Insecticide resistance has been emerging in many insect disease vectors, including mosquitoes [10,11,12,13,14]. Furthermore, the environmental impacts of synthetic insecticides have been felt for many years [15,16]. It has been reported that insecticide use has detrimental effects on non-target organisms, for example imidacloprid on honey bee (*Apis mellifera*) [17], damselfly (*Ischnura senegalensis*) [18], fathead minnow (*Pimephales promelas*), or the amphipod (*Hyalella azteca*) [19]. Thus, there is a need for new and complementary methods for controlling insect vectors, and essential oils have shown promise as renewable and environmentally-safe alternatives to the use of synthetic insecticides [20,21,22,23,24,25].

The Lamiaceae has been an important family in terms of biologically active essential oils. Essential oils from members of this family have demonstrated potential as natural insect pest control agents [24,26,27,28,29,30,31,32]. The genus *Premna* L. was formerly included in the family Verbenaceae, but has been reassigned to the Lamiaceae [33]. The genus is distributed in tropical regions of the Old World, from Africa, eastward through China, Southeast Asia and Malesia, to Australia and islands in the Pacific [34]. The number of species has been estimated to be as few as 50, or as many as 200 [34]. The ethnopharmacology, pharmacognosy, and phytochemistry of the genus have been reviewed [33,35,36,37]. As part of our ongoing efforts in identifying readily-available essential oils for mosquito control, we have examined the leaf essential oils of eight species of *Premna* (Table 1) found growing wild in central Vietnam for larvicidal activity against *Aedes aegyptae*, *Aedes albopictus*, and *Culex quinquefasciatus*. Several of these *Premna* species have been used traditionally in Vietnam (Table 1).

A perusal of the literature has revealed no previous phytochemical reports on *P. cambodiana*, *P. flavescens*, *P. maclurei*, *P. mekongensis*, or *P. puberula*.

## 2. Results and Discussion

### 2.1. Plant Collection and Essential Oils

The leaves of eight species of *Premna* were collected from several sites in Vietnam. The collection sites, voucher numbers, and essential oil yields are summarized in Table 2. 

### 2.2. Essential Oil Compositions

The *Premna* leaf essential oils were analyzed by gas chromatography–mass spectrometry and the chemical compositions are summarized in Table 3.

#### 2.2.1. *Premna cambodiana*

A total of 72 compounds were tentatively identified in the leaf essential oil of *P. cambodiana*, accounting for 97.4% of the total composition (Table 3). Sesquiterpene hydrocarbons dominated *P. cambodiana* leaf essential oil with α-copaene (23.3%), α-gurjunene (11.3%), (*E*)-caryophyllene (12.8%), and δ-cadinene (5.5%) as the major sesquiterpene components. There have been no previous phytochemical investigations on *P. cambodiana* reported in the literature; this is the first report on its essential oil composition.

#### 2.2.2. *Premna chevalieri*

Eighty-five components (99.8% of the composition) were tentatively identified in *P. chevalieri* essential oil. The major components in the leaf essential oil of *P. chevalieri* were the sesquiterpenes (*E*)-caryophyllene (31.5%) and α-humulene (7.5%) and the monoterpenes α-pinene (12.2%) and β-pinene (16.8%) (Table 3). There have been no previous phytochemical investigations on *P. chevalieri* reported in the literature; this is the first report on the leaf essential oil composition of this plant.

#### 2.2.3. *Premna corymbosa* (syn. *P. integrifolia*, *P. serratifolia*)

Leaves of *P. corymbosa* were collected from two different sites (i.e., Nậm Giải Commune, Quế Phong district, Pu Hoat Nature Reserve, Nghe An province, and Son Tra Peninsula, Da Nang province). Although the two essential oil compositions are qualitatively similar, there are notable quantitative differences (Table 3). The sample from Nghe An province was rich in oxygenated sesquiterpenoids, e.g., spathulenol (17.3%) and caryophyllene oxide (16.8%), while the sample from Da Nang was dominated by sesquiterpene hydrocarbons, including *allo*-aromadendrene (39.7%), (*E*)-caryophyllene (13.3%), and α-copaene (8.1%).

The major components of the leaf essential oil of *P. corymbosa* (reported as *P. integrifolia*) from Bangladesh were phytol (27.3%), α-humulene (14.2%), spathulenol (12.1%), 1-octen-3-ol (8.2%), eugenol (6.7%), and phenylethyl alcohol (5.8%) [46]. Neither 1-octen-3-ol, phenylethyl alcohol, nor eugenol were detected in the samples from Vietnam. Likewise, neither α-copaene nor *allo*-aromadendrene were reported from the Bangladeshi sample. In contrast, *P. corymbosa* leaf essential oil (reported as *P. serratifolia*) displayed a very simple composition of eugenol (47.9%), eugenyl acetate (9.1%), massoialactone (32.9%), and a compound identified as *cis*-2-oxabicyclo[4.4.0]decane (12.4%) (likely incorrect based on relative retention times) [47]. Thus, there is wide variation in the essential oil compositions of this plant, which suggests different chemotypes are possible or these three plants represent different species.

#### 2.2.4. *Premna flavescens*

Leaves of *P. flavescens* were collected from two different sites (i.e., Nậm Giải Commune, Quế Phong district, Pu Hoat Nature Reserve, Nghe An province, and Đồng Văn Commune, Quế Phong District, Pu Hoat Nature Reserve, Nghe An province). The leaf essential oils from the two sites showed notable differences in compositions (Table 3). (*E*)-Caryophyllene was abundant in both samples (41.0% and 11.8% in the Nậm Giải and Đồng Văn samples, respectively), as was *trans*-β-elemene (9.9% and 8.7%, respectively). The sample from Đồng Văn was rich in α-gurjunene (19.6%), but only a minor component (0.1%) in the sample from Nậm Giải. Likewise, α-guaiene and α-bulnesene were relatively abundant in the Đồng Văn sample (6.1% and 5.4%), but minor in the sample from Nậm Giải (0.5% and 0.2%, respectively). Interestingly, bicyclogermacrene (7.8%) and an unidentified component (RI 1759, 14.7%) in the sample from Nậm Giải, were not detected in the sample from Đồng Văn. Conversely, α-selinene, 8.7% in the sample from Đồng Văn, was not detected in the sample from Nậm Giải. As far as we are aware, there have been no previous reports on the essential oil chemistry of *P. flavescens*.

#### 2.2.5. *Premna maclurei*

The leaf essential oil composition of *P. maclurei* is shown in Table 3. The essential oil was dominated by sesquiterpene hydrocarbons (62.5%) and oxygenated sesquiterpenoids (30.1%) with (*E*)-caryophyllene (30.7%), α-humulene (5.3%), δ-cadinene (8.4%), spathulenol (6.8%), and caryophyllene oxide (12.3%) as the major components. To our knowledge, there have been no previous reports on the essential oil composition of *P. maclurei*.

#### 2.2.6. *Premna mekongensis*

Essential oils were obtained from leaves of *P. mekongensis* from two different locations, Ngoc Linh Nature Reserve in Quang Nam Province, and Chu Mom Ray National Park. The leaf essential oil compositions are listed in Table 3. The two samples showed very different compositions. The Ngoc Linh sample was dominated by α-pinene (66.9%) and (*E*)-caryophyllene (14.7%). The leaf essential oil from Chu Mom Ray, on the other hand, had relatively low concentrations of α-pinene (1.5%) and (*E*)-caryophyllene (3.9%). In addition, the Chu Mom Ray essential oil was much more complex with 95 identified components compared to only 37 in the Ngoc Linh sample. The high concentration of α-pinene in *P. mekongensis* leaf essential oil from Ngoc Linh was unexpected and uncharacteristic of *Premna* leaf essential oils, which are generally low in monoterpene hydrocarbon concentrations (see below). To our knowledge, there have been no previous studies on the essential oil composition of *P. mekongensis*.

#### 2.2.7. *Premna puberula*

The chemical composition of the leaf essential oil of *P. puberula* is shown in Table 3. The major chemical classes present in the essential oil were sesquiterpene hydrocarbons (22.4%), with α-copaene (5.3%) and *allo*-aromadendrene (4.1%) as major components, and oxygenated sesquiterpenoids (58.2%), dominated by (*E*)-caryophyllene oxide (21.2%) along with spathulenol (7.7%) and humulene epoxide II (4.7%). There have been no previous reports on the essential oil of *P. puberula*.

#### 2.2.8. *Premna tomentosa*

The leaf essential oil composition of *P. tomentosa* is shown in Table 3. A total of 82 compounds were tentatively identified in the essential oil accounting for 99.8% of the composition, which was dominated by sesquiterpene hydrocarbons, especially (*E*)-caryophyllene (22.0%) and germacrene D (11.4%). The only previous examination of the essential oil of *P. tomentosa* is a relatively old work by Narayan and Muthana in 1953 [48]. These workers identified limonene (57.8%), (*E*)-caryophyllene (17.2%), an unidentified cadinane sesquiterpene (7.8%), an unidentified sesquiterpene alcohol (5.6%), and an unidentified diterpene hydrocarbon (5.5%) in the leaf essential oil from southern India.

#### 2.2.9. Species Composition Comparison

Analogous to most of the *Premna* essential oil compositions observed in this study, leaf essential oils of other *Premna* species have shown compositions dominated by sesquiterpene hydrocarbons, e.g., *Premna coriacea* (55.2%) [49], *Premna latifolia* (76.4%) [50], *Premna quadrifolia* (65.5%) [51], and *Premna odorata* (62.3%) [52]. On the other hand, other *Premna* species are particularly rich in low molecular weight alcohols such as 1-octen-3-ol in *Premna barbata* (37.3%) [53], *P. latifolia* (35.7%) [54], and *Premna angolensis* (28.0%) [51]. In contrast, the essential oil of *Premna microphylla* was dominated by the sesquiterpenoid derivative blumenol C (49.7%) [55].

Compounds common to all eight of the *Premna* leaf essential oils in this study were α-pinene, β-pinene, *p*-cymene, limonene, linalool, *trans*-β-elemene, (*E*)-caryophyllene, α-humulene, β-selinene, and caryophyllene oxide. These are all relatively common essential oil constituents, and therefore cannot be considered as key compounds defining the genus. Furthermore, leaf essential oils of other *Premna* species were missing several of these components. The leaf essential oil of *P. coriacea* from Karnataka, India, was devoid of α-pinene, β-pinene, linalool, and β-selinene [49]. Likewise, the leaf oil from *P. microphylla* from Zhejiang Province, China, contained no α-pinene, β-pinene, linalool, (*E*)-caryophyllene, or α-humulene [55]. *Premna integrifolia* leaf essential oil from Bangladesh did not show *p*-cymene, limonene, linalool, β-elemene, or β-selinene [46]; *P. odorata* leaf oil from Giza, Egypt, showed no α-pinene, β-pinene, *p*-cymene, limonene, β-elemene, or β-selinene [52]; *P. angolensis* leaf oil from Comé, Benin, contained no β-pinene or caryophyllene oxide, and *P. quadrifolia* from Comé, Benin, contained no α-pinene, limonene, or linalool [51].

### 2.3. Mosquito Larvicidal Activity

The *Premna* leaf essential oils have been screened for mosquito larvicidal activity against *Aedes aegypti* and, if sufficient mosquito larvae were available, also against *Ae. albopictus* and *Culex quinquefasciatus*. The 24-h and 48-h larvicidal activities are shown in Table 4 and Table 5, respectively. Considering larvicidal activities against *Ae. aegypti*, the most active *Premna* leaf essential oils were *P. cambodiana* (24-h LC_50_ = 16.8 μg/mL) and *P. mekongensis* from Nghe An (24-h LC_50_ = 16.8 μg/mL).

The pronounced larvicidal activity of *P. mekongensis* (Ngoc Linh) against *Ae. aegypti* can be attributed to the high concentration of α-pinene. This monoterpene has shown larvicidal activity against *Ae. aegypti* with LC_50_ values ranging from 15.4 μg/mL to 79.1 μg/mL [56]. Interestingly, the larvicidal activity of *P. mekongensis* (Ngoc Linh) against *Cx. quinquefasciatus* was less (24-h LC_50_ = 42.7 μg/mL), consistent with the reduced activity of α-pinene against this mosquito larva (LC_50_ = 95 μg/mL) [57].

The major components in *P. cambodiana* leaf essential oil were the sesquiterpene hydrocarbons α-copaene (23.3%), (*E*)-caryophyllene (12.8%), and α-gurjunene (11.3%). (*E*)-Caryophyllene has shown larvicidal activity against *Ae. aegypti* with reported LC_50_ values of 38.6 μg/mL [58] and 88.3 μg/mL [59]. As far as we are aware, there have been no reports on the larvicidal activities of either α-copaene or α-gurjunene. However, essential oils rich in α-copaene have shown notable larvicidal activity. For example, the essential oil from the inflorescences of *Piper marginatum* Jacq. (Piperaceae) (9.4% α-copaene and 13.1% (*E*)-caryophyllene) showed larvicidal activity against *Ae. aegypti* with LC_50_ of 19.9 μg/mL [60]; the ripe peel essential oil of *Hymenaea courbaril* L. (Fabaceae) (11.1% α-copaene) had an LC_50_ of 14.8 μg/mL on *Ae. aegypti* [61]. Note, however, that the leaf essential oil of *P. corymbosa* from Da Nang was also rich in α-copaene (8.1%) and (*E*)-caryophyllene (13.3%), but the larvicidal activity against *Ae. aegypti* was weaker (LC_50_ = 61.8 μg/mL). Similarly, the leaf essential oil of *P. flavescens* from Đồng Văn had α-gurjunene (19.6%) and (*E*)-caryophyllene (11.8%) as major components, but also showed weak larvicidal activity against *Ae. aegypti* (LC_50_ = 64.7 μg/mL). The mere presence of the sesquiterpene hydrocarbons α-copaene, α-gurjunene, and (*E*)-caryophyllene is not sufficient to impart good larvicidal activity; there are likely synergistic effects of these compounds with minor components that account for the activities.

## 3. Materials and Methods

### 3.1. Plant Material

Leaves of *Premna* species were collected from several different locations in central Vietnam (Table 1). The plants were identified by Dr. Do Ngoc Dai, and voucher specimens (see Table 2) have been deposited in the plant specimen room, Faculty Agriculture, Forestry, and Fishery, Nghe An, College of Economics. The fresh leaves (2.0 kg each), immediately after collection, were shredded and hydrodistilled for 4 h using a Clevenger type apparatus (Witeg Labortechnik, Wertheim, Germany). Essential oil yields are summarized in Table 2. Essential oils were dried over anhydrous Na_2_SO_4_ and stored in sealed glass vials at 4 °C until analyzed.

### 3.2. Gas Chromatography–Mass Spectrometry

The *Premna* leaf essential oils were analyzed by gas chromatography–mass spectrometry (GC-MS) as described previously [56]: Shimadzu GCMS-QP2010 Ultra, electron impact (EI) mode (electron energy = 70 eV), scan range = 40–400 atomic mass units, scan rate = 3.0 scans/s; ZB-5ms column (30 m length × 0.25 mm inner diameter × 0.25 μm film thickness); He carrier gas, head pressure of 552 kPa, flow rate of 1.37 mL/min; injector temperature was 250 °C, ion source temperature was 200 °C; GC oven temperature program: 50 °C initial temperature, increased 2 °C/min to 260 °C; 5% solution of essential oil in CH_2_Cl_2_, 0.1 μL injection, splitting ratio 30:1. Putative identification of the essential oil components was based on their calculated retention indices (RI), based on a homologous series of *n*-alkanes (C_8_-C_40_), and their mass spectral fragmentation patterns compared with those reported in the databases [42,43,44,45], with RI values within ±10 units and with matching factors >80%. The concentrations of the essential oil components were calculated from raw peak areas, normalized to 100%, without standardization.

### 3.3. Mosquito Larvicidal Assay

Eggs of *Aedes aegypti* were purchased from Institute of Biotechnology, Vietnam Academy of Science and Technology and maintained at the Laboratory of Department of Pharmacy of Duy Tan University, Da Nang, Vietnam. Adults of *Culex quinquefasciatus* and *Aedes albopictus* collected in Hoa Khanh Nam ward, Lien Chieu district, Da Nang city (16°03′14.9″ N, 108°09′31.2″ E) and were identified by National institute of Malariology, Parasitology, and Entomology, Ho Chi Minh City. Adult mosquitoes were maintained in entomological cages (40 × 40 × 40 cm) and fed a 10% sucrose solution and were allowed to blood feed on 1-week-old chicks and mice, respectively. Egg hatchings were induced with tap water. Larvae were reared in plastic trays (24 × 35 × 5 cm). The larvae were fed on Koi fish food. All developmental stages were maintained at 25 ± 2 °C, 65–75% relative humidity and a 12:12 h light:dark cycle at the Laboratory of the Faculty of Environmental and Chemical Engineering of Duy Tan University, Da Nang, Vietnam.

Larvicidal activities of the *Premna* essential oils were determined following the protocol previously reported [62]: 250-mL beakers, 150 mL of water, and 20 larvae (fourth instar), aliquots of the *Premna* essential oils dissolved in EtOH (1% stock solution) were added to give final concentrations of 100, 50, 25, 12.5, and 6 μg/mL; EtOH only (negative control) and permethrin (positive control), mortality recorded after 24 h and 48 h of exposure, experiments were carried out at 25 ± 2 °C, each test was conducted with four replicates. The data obtained were subjected to log-probit analysis [63] to obtain LC_50_ values, LC_90_ values and 95% confidence limits using Minitab^®^ 19 (Minitab, LLC, State College, PA, USA).

All experimental procedures that involved animals (mice, mosquitoes, chicks, and non-target organisms) were conducted in accordance with the “Guideline for the Care and Use of Laboratory Animals” which was approved by the Medical-Biological Research Ethics Committee of Duy Tan University (DTU/REC2020/NHH01), Vietnam.

## 4. Conclusions

The leaf essential oils of eight species of *Premna* have been obtained in yields ranging from 0.10% to 0.25%. The mosquito larvicidal activities of these species have been determined for the first time and this is the first report of the essential oil compositions of *P. cambodiana*, *P. flavescens*, *P. maclurei*, *P. mekongensis*, and *P. puberula*. The essential oil compositions were largely dominated by sesquiterpene hydrocarbons and oxygenated sesquiterpenoids. The larvicidal activities against *Aedes aegypti* (LC_50_ < 65 μg/mL) are promising and can probably be attributed to these components. The essential oil yields, however, are low and likely preclude their consideration as viable alternatives to other essential oils for control of mosquito vectors. However, potential utility of these essential oils will necessitate exploration of cultivation, including plant breeding aimed at increasing oil yield and/or larvicidal activity, potential detrimental effects of the essential oils on the environment, as well as field experiments on application of the essential oils, effects of environmental conditions and potential formulations on essential oil evaporation rates.

## Figures and Tables

**Table 1:** *Premna* species examined in this study.

*Premna* Species	Native Range	Ethnobotanical Use in Vietnam
*Premna cambodiana* Dop (Vietnamese name Cách cam bốt)	Laos, Cambodia and Vietnam (Kon Tum, Gia Lai, and Đắk Nông provinces) [38,39,40].	Used to treat spermatorrhea and gynecological diseases [40].
*Premna chevalieri* Dop (syn. *Premna acuminatissima* Merr.) (Vietnamese name Cách vàng)	Thailand, Laos, Vietnam, China (Hainan, Yunnan) [41]. In Vietnam, the plant has been recorded in Thái Nguyên, Phú Thọ, Bắc Giang, Hà Nội, Hòa Bình, Ninh Bình, Nghệ An, Hà Tĩnh, and Quảng Nam provinces [39,40].	The plant is used to treat polio, jaundice [40].
*Premna corymbosa* Rottler & Willd. (syn. *Premna serratifolia* L., *Cornutia corymbosa* Burm. f., *Premna integrifolia* L., *Gumira corymbosa* (Rottler & Willd.) Kuntze) (Vietnamese name Vọng cách, Cách biển)	Ranges from Madagascar, through tropical and subtropical Asia, to Australia and Pacific islands [38]. In Vietnam, *P. corymbosa* has been found in Quảng Ninh, Hà Nội, Hải Phòng, Hà Nam, Ninh Bình, Thanh Hóa, Thừa Thiên Huế, Đà Nẵng, Quảng Nam, Khánh Hòa, Kon Tum, Đắk Nông, Đồng Nai, Hồ Chí Minh, Bà Rịa-Vũng Tàu, Long An, and Kiên Giang provinces [39,40].	The plant used to treat skin diseases. Additionally, the leaves are used as culinary additives [40].
*Premna flavescens* Buch.-Ham. ex C.B. Clarke (syn. *Premna lucidula* Miq.)	Southern China (Guangdong, Guangxi, and southern Yunnan), India, Indonesia, Malaysia, and Vietnam [41]. In Vietnam, *P. flavescens* has been recorded in Vĩnh Phúc, Nghệ An, Quảng Nam, Kon Tum, Gia Lai, Đắk Nông, and Đồng Nai provinces [39,40].	A species commonly grown in Vietnam; a decoction of the leaves is taken daily as a tonic [40].
*Premna maclurei* Merr. (Vietnamese name Cách maclura)	China (Hainan) [41] as well as the provinces of Nghệ An and Quảng Nam, Vietnam [39,40].	
*Premna mekongensis* W.W. Sm. (Vietnamese name Cách mê công)	China (northwestern and western Yunnan province) [41] and in Vietnam (Hà Giang and Quảng Nam Provinces) [39,40].	
*Premna puberula* Pamp. (syn. *Premna martini* H.Lév.) (Vietnamese name Cách lún phún)	China (Fujian, Gansu, Guangdong, Guangxi, Guizhou, Hubei, Hunan, southern Shanxi, Sichuan, and Yunnan) [41] as well as Vietnam (Hà Giang, Bắc Giang, and Nghệ An) [39,40].	Used in traditional medicine [40].
*Premna tomentosa* Willd. (syn. *Premna cordata* Blanco) (Vietnamese name Cách lông tơ)	Ranges from China (Guangdong), through tropical Asia, to North Queensland, Australia [38]. In Vietnam, the plant has been recorded in Nghệ An province and South Vietnam [39,40].	Leaves, roots as medicine [40].

**Table 2:** Collection details and yields for *Premna* leaf essential oils from central Vietnam.

*Premna* Species	Collection Site	Voucher Numbers	Essential Oil Yield (% *v*/*w*)
*Premna cambodiana*	Chu Mom Ray National Park 14°25′33.5″ N, 107°43′15.6″ E, 672 m elevation	DND 88	0.14
*Premna chevalieri*	Tay Giang District, Quang Nam Province 15°49′59″ N 107°21′10″ E, 962 m elevation	DND 101	0.10
*Premna corymbosa*	Nậm Giải Commune, Quế Phong district, Pu Hoat Nature Reserve, Nghe An province 19°41′40″ N, 104°49′29″ E, 670 m elevation	DND 788	0.22
	Son Tra Peninsula, Da Nang province 16°05′57″ N 108°15′59″ E, 6 m elevation	DND 49	0.25
*Premna flavescens*	Nậm Giải Commune, Quế Phong district, Pu Hoat Nature Reserve, Nghe An province 19°41′40″ N, 104°49′29″ E, 670 m elevation	DND 787	0.11
	Đồng Văn Commune, Quế Phong District, Pu Hoat Nature Reserve, Nghe An province 19°50′45″ N, 105°06′09″ E, 511 m elevation	DND 711	0.12
*Premna maclurei*	Nậm Giải Commune, Quế Phong district, Pu Hoat Nature Reserve, Nghe An province 19°41′40″ N, 104°49′29″ E, 670 m elevation	DND 747	0.12
*Premna mekongensis*	Ngoc Linh Nature Reserve, Quang Nam Province 15°50′16.0″ N, 107°22′54.7″ E, 1341 m elevation	DND 102	0.19
	Chu Mom Ray National Park 14°25′33.5″ N, 107°43′15.6″ E, 672 elevation	DND 84	0.21
*Premna puberula*	Đồng Văn Commune, Quế Phong District, Pu Hoat Nature Reserve, Nghe An province 19°50ʹ45″ N, 105°06′09″ E, 511 m elevation	DND 710	0.11
*Premna tomentosa*	Nghia Dan District, Nghe An province 19°20′23″ N 105°25′18″ E, 49 elevation	DND 23	0.12

**Table 3:** Chemical compositions of leaf essential oils of *Premna* species from central Vietnam.

RI_calc_^a^	RI_db_^b^	Compound ^c^	*P. cambodiana*	*P. chevalier*	*P. corymbosa* (Nghe An)	*P. corymbosa* (Da Nang)	*P. flavescens* (Nậm Giải)	*P. flavescens* (Đồng Văn)	*P. maclurei*	*P. mekongensis* (Ngoc Linh)	*P. mekongensis* (Chu Mom Ray)	*P. puberula*	*P. tomentosa*
922	923	Tricyclene	---	tr ^d^	---	---	---	---	---	---	tr	---	---
925	927	α-Thujene	---	0.1	0.1	---	---	---	---	---	tr	---	0.1
931	933	α-Pinene	1.9	12.2	0.5	0.5	0.2	0.1	0.4	66.9	1.5	1.3	3.0
947	948	α-Fenchene	---	tr	---	---	---	---	---	---	---	---	---
949	953	Camphene	---	0.1	---	---	tr	tr	---	0.2	1.5	---	tr
952	953	Thuja-2,4(10)-diene	---	tr	---	---	---	---	---	0.1	---	---	---
971	971	Sabinene	---	tr	0.8	tr	---	tr	tr	0.9	0.1	---	2.0
976	978	β-Pinene	1.7	16.8	0.6	0.1	0.1	0.1	0.2	0.5	0.7	0.1	0.7
978	978	1-Octen-3-ol	---	---	---	---	---	0.3	---	0.2	---	---	tr
978	986	6-Methylhept-5-en-2-one	---	---	---	---	---	---	tr	---	---	---	---
983	986	3-Octanone	---	0.1	---	---	---	---	---	---	---	---	---
987	991	Myrcene	---	0.2	---	0.4	---	0.3	tr	1.6	3.1	---	0.1
997	999	3-Octanol	---	0.1	---	---	---	---	---	---	---	---	tr
1001	1004	*p*-Mentha-1(7),8-diene	---	---	---	tr	---	---	---	---	---	---	---
1006	1007	α-Phellandrene	---	---	---	0.2	---	---	---	0.3	1.6	---	tr
1008	1009	δ-3-Carene	---	---	---	---	---	tr	---	---	---	---	---
1017	1018	α-Terpinene	---	tr	---	---	---	---	---	---	tr	---	0.1
1023	1025	*p*-Cymene	tr	0.1	0.5	1.9	0.1	tr	tr	0.3	0.6	0.1	0.4
1027	1030	Limonene	0.2	1.0	0.2	1.7	0.1	0.1	0.1	1.1	1.7	0.3	0.2
1029	1031	β-Phellandrene	---	tr	---	0.6	tr	---	---	0.4	1.3	---	tr
1031	1032	1,8-Cineole	---	tr	0.1	---	0.1	tr	---	0.9	tr	---	tr
1034	1034	(*Z*)-β-Ocimene	---	0.1	---	---	---	---	---	0.1	tr	---	tr
1045	1045	(*E*)-β-Ocimene	---	1.3	---	---	---	tr	---	0.1	0.2	---	0.1
1057	1057	γ-Terpinene	---	tr	---	---	---	---	---	0.1	tr	---	0.2
1085	1086	Terpinolene	---	tr	---	---	---	---	---	tr	0.1	---	0.1
1098	1101	Linalool	tr	0.8	0.8	0.2	0.1	0.4	0.2	1.4	0.3	0.1	0.2
1102	1104	2-Methylbutyl 2-methylbutanoate	---	---	---	---	---	---	---	---	tr	---	---
1103	1104	Nonanal	tr	0.1	---	tr	tr	---	tr	0.1	---	---	---
1107	1108	*p*-Mentha-2,8-dien-1-ol	---	---	---	---	---	---	---	---	---	0.4	---
1111	1113	(3*E*)-4,8-Dimethyl-1,3,7-nonatriene	---	1.1	---	---	---	---	tr	---	---	---	tr
1125	1126	α-Campholenal	---	---	---	---	---	---	---	---	---	0.1	---
1136	1138	Benzeneacetonitrile	---	---	---	---	---	tr	---	---	---	0.1	---
1139	1141	*trans*-Pinocarveol	tr	0.1	---	---	---	---	---	---	---	0.2	---
1143	1145	*trans*-Verbenol	---	---	---	---	---	---	---	0.1	---	0.3	---
1146	1153	*p*-Vinylanisole	---	---	---	---	---	---	0.1	---	---	---	0.2
1161	1164	Pinocarvone	---	tr	---	---	---	---	---	---	---	---	---
1170	1173	Borneol	---	---	---	---	---	---	---	---	tr	---	---
1179	1180	Terpinen-4-ol	---	---	0.1	---	---	---	tr	---	tr	---	0.3
1182	1187	(3*Z*)-Hexenyl butyrate	---	---	---	tr	---	0.1	---	---	---	---	---
1182	1188	Naphthalene	---	---	---	---	---	0.1	---	---	---	---	---
1184	1187	Cryptone	---	---	---	tr	---	---	---	---	tr	---	---
1185	1188	*p*-Cymen-8-ol	---	---	---	---	---	---	---	---	---	0.1	---
1189	1193	Butyl hexanoate	---	---	---	---	---	---	---	---	0.6	---	---
1189	1192	Methyl salicylate	---	0.1	---	tr	---	---	tr	---	---	---	tr
1192	1196	Myrtenal	tr	---	---	---	---	---	tr	---	---	0.1	---
1193	1195	α-Terpineol	---	0.1	---	tr	---	---	tr	0.1	tr	---	tr
1200	1202	*cis*-Sabinol	---	---	---	0.1	---	---	---	---	tr	---	---
1204	1208	Decanal	tr	---	---	---	---	---	---	---	---	---	---
1205	1208	Verbenone	---	---	---	---	---	---	---	---	---	0.1	---
1206	1207	(3*E*)-Octenyl acetate	---	0.1	---	---	---	---	---	---	---	---	---
1227	1229	Thymol methyl ether	---	---	---	---	---	---	---	---	0.1	---	---
1247	1257	*p*-Anisaldehyde	---	---	---	---	---	---	tr	---	---	---	---
1248	1250	Linalyl acetate	---	---	---	---	---	---	---	---	tr	---	---
1251	1252	Isopentyl hexanoate	---	---	---	---	---	---	---	---	0.3	---	---
1282	1282	Bornyl acetate	---	tr	---	---	tr	---	---	---	3.9	---	---
1286	1287	Dihydroedulan IA	---	tr	---	---	0.8	---	tr	---	0.2	---	tr
1291	1294	Dihydroedulan IIA	---	0.1	---	---	---	tr	0.4	---	---	---	0.1
1297	1299	Theaspirane A	---	tr	---	---	---	---	---	---	---	---	---
1300	1300	Tridecane	---	---	---	---	tr	---	---	---	---	---	---
1307	1306	Isoascaridole	---	---	---	---	---	---	---	---	---	---	tr
1312	-	Unidentified ^e^	---	---	---	---	---	1.2	---	---	3.4	---	---
1313	1315	Theaspirane B	---	tr	---	---	---	---	---	---	---	---	---
1317	1318	3-Hydroxycineole	---	---	---	---	---	---	---	---	tr	---	---
1329	1328	Bicycloelemene	---	---	---	0.1	0.6	0.1	---	---	1.1	---	0.2
1332	1335	δ-Elemene	0.2	---	---	---	0.1	tr	0.2	---	0.6	---	0.2
1345	1349	α-Cubebene	0.7	0.1	0.5	0.3	---	0.2	0.5	---	0.4	0.5	0.2
1345	1349	α-Terpinyl acetate	---	---	---	---	---	---	tr	---	1.5	---	---
1356	1361	Neryl acetate	---	---	---	---	---	---	---	---	tr	---	---
1362	1367	Decanoic acid	---	tr	---	---	---	---	---	---	---	---	---
1365	1372	Isoledene	---	---	---	---	---	---	tr	---	---	---	---
1367	1371	α-Ylangene	0.3	---	0.6	0.1	---	---	0.1	---	0.3	---	0.1
1368	1367	Cyclosativene	tr	---	---	---	tr	0.1	---	---	0.1	---	---
1374	1378	Geranyl acetate	0.1	---	---	---	---	---	---	---	---	---	---
1374	1375	α-Copaene	**23.3**	0.9	**6.8**	**8.1**	0.1	2.9	2.6	---	1.6	**5.3**	3.1
1376	1379	(*E*)-β-Damascenone	---	0.1	---	---	---	---	0.1	---	---	---	tr
1378	1382	(3*Z*)-Hexenyl hexanoate	---	tr	---	---	---	---	---	---	---	---	---
1379	1383	*cis*-β-Elemene	0.2	tr	---	tr	0.5	0.5	0.1	---	0.1	---	0.3
1381	1382	β-Bourbonene	tr	tr	---	tr	---	0.1	0.1	---	---	---	tr
1384	1390	Hexyl hexanoate	---	---	---	---	---	---	---	---	2.1	---	---
1386	1392	β-Cubebene	1.1	0.2	1.8	0.9	---	0.6	1.6	---	0.3	1.5	0.7
1387	1390	*trans*-β-Elemene	3.5	0.3	1.5	1.0	**9.9**	**8.7**	1.8	0.5	1.3	1.9	**5.0**
1402	1405	(*Z*)-Caryophyllene	---	0.1	---	---	0.2	---	---	---	---	---	---
1402	1403	9,10-Dehydroisolongifolene	---	---	---	---	---	---	---	---	---	0.8	---
1402	1406	Cyperene	---	---	---	---	---	1.1	---	---	0.1	---	---
1405	1406	α-Gurjunene	**11.3**	---	0.6	0.1	0.1	**19.6**	---	---	0.3	1.3	**5.2**
1412	1415	β-Maaliene	0.2	---	---	---	---	0.4	---	---	---	---	2.8
1419	1417	(*E*)-Caryophyllene	**12.8**	**31.5**	**6.9**	**13.3**	**41.0**	**11.8**	**30.7**	**14.7**	3.9	0.6	**22.0**
1427	1427	γ-Elemene	0.7	tr	---	---	---	---	---	---	---	---	2.4
1427	1430	γ-Maaliene	---	---	---	---	---	---	---	---	0.1	---	0.3
1428	1433	β-Copaene	---	tr	0.2	0.2	---	0.1	0.4	---	1.2	---	---
1429	1430	*trans*-α-Bergamotene	---	tr	---	0.1	0.7	---	---	---	---	---	---
1430	1437	β-Gurjunene (= Calarene)	---	---	---	---	---	0.1	---	---	---	---	---
1432	1436	α-Guaiene	0.1	0.1	---	4.6	0.5	**6.1**	0.4	0.5	---	0.4	0.3
1433	1438	α-Maaliene	0.1	---	---	---	---	---	---	---	0.1	---	---
1437	1438	Aromadendrene	0.1	tr	0.5	0.1	0.4	0.1	0.3	---	1.0	---	3.4
1443	1444	Guaia-6,9-diene	0.1	---	---	---	1.1	tr	---	---	---	---	0.1
1444	1445	Selina-5,11-diene	---	0.1	---	---	---	---	---	---	0.1	---	---
1445	1446	Neryl acetone	---	tr	---	---	---	---	---	---	---	---	---
1448	1453	*trans*-Muurola-3,5-diene	---	---	---	---	---	---	---	---	0.1	---	---
1448	1445	β-Barbatene	---	tr	---	---	---	---	---	---	---	---	---
1450	1447	*iso*-Germacrene D	---	---	---	---	---	---	---	---	---	---	tr
1450	1455	Valerena-4,7(11)-diene	0.1	---	---	0.4	---	0.3	---	---	0.1	---	0.1
1451	1452	(*E*)-β-Farnesene	---	0.1	---	---	0.1	---	---	---	---	---	---
1454	1454	α-Humulene	3.7	**7.5**	2.6	3.7	2.4	3.1	**5.3**	2.5	2.0	0.4	3.9
1458	1457	*allo*-Aromadendrene	1.6	0.1	**7.7**	**39.7**	0.1	3.0	0.2	---	1.0	4.1	1.0
1458	1463	*cis*-Cadina-1(6),4-diene	0.1	---	---	---	---	---	---	---	---	---	---
1461	1459	Rotundene	---	---	---	---	---	0.1	---	---	---	---	---
1462	1466	*cis*-Muurola-4(14),5-diene	---	---	---	---	---	---	---	---	0.2	---	0.1
1469	1473	4,5-di-*epi*-Aristolochene	---	0.1	1.0	---	---	0.1	---	---	---	---	---
1471	1472	*trans*-Cadina-1(6),4-diene	---	---	---	---	---	---	---	---	0.4	---	---
1472	1478	γ-Muurolene	0.6	---	0.4	0.4	0.1	---	1.2	---	1.7	---	0.4
1473	1476	γ-Gurjunene	0.8	---	---	---	---	0.4	---	---	0.3	---	0.3
1474	1475	Selina-4,11-diene	---	2.0	---	---	0.2	0.1	---	0.5	---	---	---
1475	1477	β-Chamigrene	---	---	---	---	---	1.3	0.1	---	---	---	---
1476	1481	(*E*)-β-Ionone	---	tr	---	---	---	---	---	---	---	---	---
1476	1479	α-Amorphene	0.3	---	---	0.2	---	---	---	---	0.6	---	---
1479	1480	Germacrene D	2.2	0.3	0.4	3.0	0.7	3.8	3.0	0.1	**5.6**	---	**11.4**
1479	1482	γ-Himachalene	0.1	---	---	---	---	---	---	---	---	---	---
1480	1483	*trans*-β-Bergamotene	---	---	---	---	0.2	---	---	---	---	---	---
1484	1491	Eremophilene	---	---	---	---	0.1	---	---	---	---	---	---
1486	1488	δ-Selinene	---	---	---	---	---	---	---	---	0.2	---	---
1487	1487	β-Selinene	1.4	1.2	4.0	0.2	1.3	**9.7**	0.3	0.4	0.7	0.7	4.3
1491	1492	*trans*-Muurola-4(14),5-diene	---	---	---	---	---	---	---	---	1.7	---	---
1491	1492	Valencene	---	---	0.2	---	---	---	0.2	---	---	---	---
1491	1491	Viridiflorene (= Ledene)	0.1	---	---	0.3	0.1	1.8	---	---	---	---	---
1491	1490	γ-Amorphene	0.2	---	---	---	---	---	---	---	---	---	0.3
1493	1493	Curzerene	---	---	---	---	---	---	---	---	1.2	---	---
1493	1498	*epi*-Cubebol	---	---	---	---	---	---	---	---	---	0.9	---
1495	1497	α-Muurolene	0.5	---	0.2	0.3	0.2	---	0.4	---	---	---	0.2
1495	1497	α-Selinene	1.0	1.5	2.7	---	---	**8.7**	---	0.4	---	---	**5.5**
1496	1497	Bicyclogermacrene	---	---	---	1.1	**7.8**	---	1.9	---	**11.9**	---	---
1497	1502	ε-Amorphene	---	0.1	---	---	---	---	---	---	---	---	---
1499	1505	α-Bulnesene	---	0.1	---	4.1	0.2	**5.4**	0.3	0.1	---	0.6	0.1
1502	1503	(*E*,*E*)-α-Farnesene	---	3.4	---	---	0.3	---	---	0.1	---	---	1.0
1503	1508	β-Bisabolene	---	---	---	0.4	0.5	---	---	---	---	---	---
1508	1511	Germacrene A	---	---	---	---	---	0.2	---	---	---	---	0.1
1511	1512	γ-Cadinene	0.4	tr	0.2	0.3	0.1	0.2	0.4	---	1.1	---	0.2
1513	1515	Cubebol	0.1	---	0.6	---	---	---	0.2	---	0.3	0.6	tr
1516	1518	δ-Cadinene	**5.5**	0.3	0.4	2.0	0.1	0.9	**8.4**	---	3.2	---	1.8
1518	1518	7-*epi*-α-Selinene	---	---	0.2	---	---	---	---	---	---	---	---
1518	1521	α-Panasinsen	---	---	---	---	---	0.5	---	---	---	---	---
1519	1519	*trans*-Calamenene	0.5	0.1	0.4	0.3	---	---	0.3	---	1.0	---	0.1
1522	1521	Zonarene	---	---	---	---	---	---	---	---	0.2	---	---
1532	1538	α-Cadinene	0.2	---	---	0.1	---	---	0.1	---	0.3	---	---
1534	1536	*trans*-Cadina-1,4-diene	---	---	---	---	---	---	---	---	0.2	---	0.1
1535	1529	*cis*-Calamenene	---	---	---	---	---	---	0.2	---	---	---	---
1536	1540	(*E*)-α-Bisabolene	---	---	---	---	0.3	---	---	---	---	---	---
1538	1540	Selina-4(15),7(11)-diene	---	---	---	---	---	---	---	---	---	---	0.1
1539	1546	*cis*-Sesquisabinene hydrate	---	---	---	---	---	---	---	---	---	---	0.1
1539	1544	α-Calacorene	0.9	---	0.6	0.1	---	---	0.6	---	0.2	3.4	---
1546	1546	α-Elemol	---	0.1	---	---	---	---	---	---	0.2	---	---
1547	1551	(*Z*)-Caryophyllene oxide	0.4	---	---	---	---	0.1	0.6	---	---	1.5	---
1550	1549	*cis*-Muurol-5-en-4β-ol	---	---	---	---	---	---	---	---	0.1	---	---
1557	1557	Germacrene B	0.6	---	---	---	0.1	tr	---	---	0.5	---	3.5
1559	1560	(*E*)-Nerolidol	---	0.2	---	---	0.3	---	---	0.1	**7.5**	---	---
1560	1560	β-Calacorene	0.4	---	---	---	---	---	0.7	---	---	0.8	---
1563	1558	1-Tetradecanol	---	0.3	---	---	---	---	---	---	---	---	---
1565	1566	1,5-Epoxysalvial-4(14)-ene	---	---	---	---	---	---	---	---	---	1.1	---
1568	1568	Palustrol	0.3	---	---	---	---	0.6	**6.8**	---	1.7	1.9	---
1569	1571	Cedroxyde	---	---	---	0.1	---	---	---	---	---	0.9	---
1572	1568	Dendrolasin	---	2.0	---	---	---	---	---	---	---	---	---
1575	1578	Furopelargone B	---	---	---	---	0.2	---	---	---	---	---	---
1575	1576	Spathulenol	0.8	0.6	**17.3**	1.4	1.8	1.0	---	---	2.8	**7.7**	0.8
1580	1587	(*E*)-Caryophyllene oxide	4.3	**5.3**	**16.8**	3.0	1.7	1.3	**12.3**	0.8	0.3	**21.2**	1.1
1584	1590	Globulol	0.5	0.1	1.7	0.3	0.3	0.2	0.3	---	1.2	2.0	0.7
1590	1596	Cubeban-11-ol	---	---	---	0.1	---	---	---	---	---	---	---
1593	1594	Viridiflorol	---	---	1.8	0.3	0.2	0.1	---	---	**5.6**	0.7	---
1595	1593	Guaiol	---	1.2	---	---	---	---	---	---	---	---	---
1598	1600	Curzerenone	---	---	---	---	---	---	---	---	0.3	---	---
1602	1605	Ledol	0.2	---	0.3	0.3	---	0.3	---	---	1.2	0.6	**6.1**
1607	1613	Humulene epoxide II	0.8	1.0	3.4	0.4	0.1	0.2	1.0	0.1	---	4.7	0.1
1612	1615	Rosifoliol	---	1.1	---	---	---	---	---	---	0.1	---	---
1614	1616	1,10-di-*epi*-Cubenol	---	---	---	---	---	---	---	---	0.2	---	---
1622	1632	Muurola-4,10(14)-dien-1β-ol	0.1	---	---	---	---	---	0.2	---	---	---	---
1624	1627	1-*epi*-Cubenol	0.4	---	0.4	0.2	---	---	0.1	---	1.0	---	---
1629	1629	*iso*-Spathulenol	---	---	1.0	0.1	0.4	---	0.5	---	---	---	0.1
1633	1635	Caryophylla-4(12),8(13)-dien-5β-ol	0.1	---	---	---	0.2	---	0.6	0.1	---	---	---
1633	1634	*cis*-Cadin-4-en-7-ol	0.2	---	---	---	---	---	---	---	0.9	---	---
1637	1641	*allo*-Aromadendrene epoxide	0.6	---	---	0.3	---	0.1	---	---	0.1	---	0.2
1641	1643	τ-Cadinol	---	---	0.4	0.2	---	---	---	---	---	---	---
1642	1643	Cubenol	---	---	---	---	---	---	---	---	1.5	---	---
1642	1643	Hedycariol	---	0.1	---	---	---	---	---	---	---	---	---
1643	1643	α-Muurolol (= δ-Cadinol)	0.3	---	0.3	0.1	0.1	---	---	---	0.4	---	0.1
1643	1645	τ-Murrolol	0.2	---	0.4	0.2	---	---	---	---	---	---	0.1
1647	1649	β-Eudesmol	---	---	---	---	---	---	0.1	---	---	---	---
1653	1652	α-Cadinol	0.6	0.3	1.9	0.3	0.3	0.1	0.3	---	1.6	0.6	0.3
1654	1663	*cis*-Calamenen-10-ol	0.4	---	---	---	---	---	0.9	---	---	0.8	---
1654	1653	Pogostol	---	0.3	0.3	---	---	---	0.8	1.4	---	1.2	0.4
1657	1658	Selin-11-en-4α-ol	0.3	---	0.9	---	0.4	0.2	---	---	0.3	---	---
1659	1664	*ar*-Turmerone	---	---	---	---	---	---	---	0.1	---	---	---
1662	1670	*trans*-Calamenen-10-ol	0.6	---	---	---	---	---	0.7	---	---	1.0	---
1663	1664	Bulnesol	---	0.4	---	---	---	---	---	---	---	---	---
1664	1662	9-Methoxycalamenene	---	---	0.7	tr	---	---	---	---	---	---	---
1668	1677	Cadalene	0.3	---	---	---	---	---	---	---	---	---	---
1669	1668	14-Hydroxy-9-*epi*-(*E*)-caryophyllene	---	---	0.5	---	---	---	---	---	---	---	---
1671	1681	Mustakone	---	---	---	---	---	---	3.6	---	---	2.6	---
1674	1677	Apiole	---	---	1.6	---	0.6	---	---	---	---	---	---
1682	1683	Germacra-4(15),5,10(14)-trien-1α-ol	---	---	1.0	---	---	---	---	---	---	---	---
1685	1688	α-Bisabolol	---	0.1	---	---	---	---	---	---	---	---	---
1692	1701	10-*nor*-Calamenen-10-one	---	---	---	---	---	---	0.4	---	---	---	---
1699	1704	*cis*-Thujopsenol	---	---	---	0.1	---	---	---	---	---	1.8	---
1700	1708	δ-Dodecalactone	---	---	---	---	---	0.1	---	---	---	---	---
1727	1729	Zerumbone	---	---	---	---	---	0.4	---	---	---	1.1	---
1744	1746	α-Cyperone	---	---	0.6	---	---	---	---	---	---	---	---
1746	1748	Geranyl hexanoate	---	---	---	---	---	---	---	---	0.2	---	---
1748	1763	β-Costol	---	---	---	0.4	---	---	---	---	---	---	---
1749	1757	Cyclocolorenone	1.1	---	---	---	---	0.1	---	---	---	2.0	---
1758	1768	Squamulosone	---	---	---	---	---	---	---	---	---	0.6	---
1759	-	Unidentified ^f^	---	---	---	---	**14.7**	---	---	---	---	---	---
1761	-	Unidentified ^g^	---	---	---	---	2.0	---	---	---	---	---	---
1806	1813	Nootkatone	---	---	0.5	0.3	---	---	---	---	---	1.4	---
1830	1836	Neophytadiene	---	---	---	---	0.1	---	---	---	---	---	---
1837	1841	Phytone	0.2	---	---	---	0.1	---	0.3	---	---	1.9	---
1859	1860	Platambin	---	---	---	---	---	---	0.8	---	---	---	0.1
1873	1879	4-Phytadiene	---	---	---	---	0.1	---	---	---	---	---	---
1886	1884	Corymbolone	---	---	---	---	---	---	---	---	---	1.1	---
1939	1947	*iso*-Phytol	---	---	---	---	0.1	---	---	---	---	---	---
1958	1958	(*Z*,*Z*)-Geranyl linalool	---	0.1	---	---	---	---	---	---	---	---	---
1983	1995	Manool oxide	---	---	---	---	---	---	0.4	---	---	---	---
2019	2022	(*E*,*E*)-Geranyl linalool	---	0.1	---	---	---	---	---	---	---	---	---
2102	2102	(*E*)-Phytol	4.3	2.2	---	0.3	4.9	---	2.4	2.2	---	---	0.7
2131	2138	Palmitaldehyde, diallyl acetal	0.6	---	---	---	---	---	0.4	---	---	---	---
		Monoterpene hydrocarbons	3.8	31.8	2.7	5.3	0.5	0.6	0.7	72.5	12.5	1.8	7.0
		Oxygenated monoterpenoids	0.1	1.0	1.0	0.2	0.2	0.4	0.2	2.5	6.0	1.4	0.5
		Sesquiterpene hydrocarbons	76.1	49.9	40.2	85.4	69.9	92.2	62.5	19.6	46.9	22.4	81.0
		Oxygenated sesquiterpenoids	12.3	12.6	50.6	8.2	6.0	4.8	30.1	2.5	27.4	58.2	10.2
		Diterpenoids	4.5	2.4	0.0	0.3	5.2	0.0	3.1	2.2	0.0	1.9	0.7
		Others	0.6	2.0	1.6	tr	1.4	0.6	1.1	0.3	3.2	0.1	0.3
		Total Identified	97.4	99.8	96.1	99.6	83.3	98.6	97.7	99.6	96.0	85.7	99.8

^a^ RI_calc_ = Retention indices determined with respect to a homologous series of *n*-alkanes on a ZB-5ms column. ^b^ RI_db_ = Retention indices from the databases [42,43,44,45]. ^c^ Tentative identification based on RI and MS fragmentation agreement. ^d^ tr = Trace (<0.05%). ^e^ MS: 162(42%), 147(54%), 133(24%), 120(24%), 119(36%), 105(100%), 91(79%), 79(37%), 77(26%), 65(14%), 55(23%), and 41(21%). ^f^ MS: 204(28%), 147(5%), 134(12%), 133(100%), 120(45%), 107(41%), 105(16%), 91(8%), 77(11%), 55(7%), and 41(6%). ^g^ MS: 206(10%), 107(100%), 77(6%), and 41(3%). Concentrations of major components are highlighted in bold.

**Table 4:** Twenty-four-hour mosquito larvicidal activities of *Premna* leaf essential oils.

*Premna* Species (Collection Site)	LC_50_ (95% Limits), μg/mL	LC_90_ (95% Limits), μg/mL	χ^2^	*p*
	*Aedes aegypti*			
*P. cambodiana* (Chu Mom Ray)	16.79 (14.66–18.68)	28.02 (25.18–32.82)	0.00624	0.997
*P. chevalieri* (Quang Nam)	31.72 (29.20–34.48)	46.88 (43.14–52.00)	2.54	0.281
*P. corymbosa* (Nghe An)	37.96 (33.16–43.18)	75.43 (66.72–88.43)	4.53	0.104
*P. corymbosa* (Da Nang)	61.78 (57.16–67.71)	83.01 (75.71–93.93)	5.75	0.056
*P. flavescens* (Đồng Văn)	64.67 (58.99–71.10)	106.1 (95.9–120.2)	13.23	0.001
*P. maclurei* (Nghe An)	43.66 (40.67–47.07)	60.72 (56.03–67.58)	1.211	0.546
*P. mekongensis* (Ngoc Linh)	17.98 (14.79–20.71)	35.81 (31.76–42.26)	4.14	0.126
*P. mekongensis* (Chu Mom Ray)	41.63 (38.79–44.49)	55.94 (52.45–60.49)	35.0	0.000
*P. puberula* (Nghe An)	50.88 (46.25–56.36)	80.60 (72.74–91.86)	12.7	0.002
*P. tomentosa* (Nghe An)	34.21 (31.02–37.67)	54.36 (49.42–61.35)	0.225	0.893
Permethrin (control)	0.0094 (0.0082–0.0107)	0.0211 (0.0185–0.0249)	57.6	0.000
	*Aedes albopictus*			
*P. corymbosa* (Da Nang)	45.89 (42.61–49.88)	64.70 (59.15–73.12)	1.55	0.460
*P. flavescens* (Đồng Văn)	90.02 (80.92–99.87)	165.4 (148.9–189.2)	4.51	0.105
*P. puberula* (Nghe An)	115.9 (108.2–124.1)	176.7 (165.0–191.8)	12.2	0.007
Permethrin (control)	0.0024 (0.0021–0.0026)	0.0042 (0.0038–0.0049)	4.64	0.031
	*Culex quinquefasciatus*			
*P. chevalieri* (Quang Nam)	75.68 (68.51–84.52)	129.8 (115.9–150.0)	6.94	0.031
*P. mekongensis* (Ngoc Linh)	42.66 (38.71–47.43)	69.35 (62.21–79.95)	1.68	0.431
*P. mekongensis* (Chu Mom Ray)	33.16 (30.30–36.25)	52.01 (47.55–58.29)	11.8	0.003
*P. puberula* (Nghe An)	60.59 (55.77–66.33)	87.68 (80.11–98.09)	12.4	0.002
Permethrin (control)	0.0188 (0.0173–0.0206)	0.0294 (0.0270–0.0326)	24.1	0.000

**Table 5:** Forty-eight-hour mosquito larvicidal activities of *Premna* leaf essential oils.

*Premna* Species (Collection Site)	LC_50_ (95% Limits), μg/mL	LC_90_ (95% Limits), μg/mL	χ^2^	*p*
	*Aedes aegypti*			
*P. cambodiana* (Chu Mom Ray)	13.68 (10.72–15.77)	25.62 (22.82–30.59)	0.00399	0.998
*P. chevalieri* (Quang Nam)	30.23 (27.75–32.92)	45.11 (41.41–50.23)	4.59	0.101
*P. corymbosa* (Nghe An)	33.59 (28.68–38.65)	71.64 (62.98–84.86)	2.98	0.225
*P. corymbosa* (Da Nang)	60.43 (55.81–66.17)	83.54 (76.24–94.13)	8.07	0.018
*P. flavescens* (Đồng Văn)	62.42 (56.58–69.12)	105.9 (96.5–119.0)	2.33	0.312
*P. maclurei* (Nghe An)	41.63 (38.85–44.63)	57.07 (53.07–62.68)	0.922	0.631
*P. mekongensis* (Ngoc Linh)	17.62 (15.37–19.67)	30.00 (26.76–35.65)	0.0364	0.982
*P. mekongensis* (Chu Mom Ray)	38.70 (36.18–41.21)	49.94 (47.01–53.73)	0.130	0.937
*P. puberula* (Nghe An)	45.71 (41.21–50.97)	76.15 (68.30–87.56)	3.40	0.182
*P. tomentosa* (Nghe An)	31.4 (28.32–34.69)	50.80 (46.13–57.36)	0.0878	0.957
Permethrin (control)	0.0087 (0.0074–0.0102)	0.0204 (0.0181–0.0236)	39.6	0.000
	*Aedes albopictus*			
*P. corymbosa* (Da Nang)	35.13 (31.93–38.74)	56.97 (51.54–64.86)	0.148	0.929
*P. flavescens* (Đồng Văn)	74.14 (66.55–81.95)	133.2 (121.1–149.9)	9.87	0.007
*P. puberula* (Nghe An)	98.1 (91.0–105.7)	151.1 (140.3–165.0)	37.2	0.000
	*Culex quinquefasciatus*			
*P. chevalieri* (Quang Nam)	52.10 (44.16–60.92)	121.1 (104.4–147.9)	6.65	0.036
*P. mekongensis* (Ngoc Linh)	38.72 (34.62–43.45)	68.87 (61.30–80.21)	0.584	0.747
*P. mekongensis* (Chu Mom Ray)	27.02 (23.51–30.51)	51.55 (45.96–60.01)	3.55	0.169
*P. puberula* (Nghe An)	41.31 (37.07–46.27)	72.04 (64.28–83.52)	2.20	0.333
